# Vitamin D binding protein gene polymorphisms and the risk of respiratory diseases: a meta-analysis and systematic review

**DOI:** 10.1186/s12920-026-02443-1

**Published:** 2026-08-13

**Authors:** Jing Zhang, Hongwen Li, Xiangyu Chi, Guoying Zhang, Hongjuan Jiang

**Affiliations:** 1 Department of Geriatric Respiratory Disease, Shandong Provincial Hospital Affiliated to Shandong First Medical University, Jinan, China; 2 Department of Geriatrics, Shandong Provincial Hospital Affiliated to Shandong First Medical University, Jinan, China

**Keywords:** Vitamin D binding protein, Respiratory disease, Gene polymorphism, Meta-analysis

## Abstract

**Background:**

Recent studies have found that VDBP gene polymorphism is closely related to the pathogenesis of some respiratory diseases. Rs7041 and rs4588 are two major single-nucleotide polymorphisms(SNPs) in the GC gene encoding VDBP. Different combinations of these variants give rise to three major haplotypic isoforms, GC*1F, GC*1S, and GC*2, which may influence pulmonary immune-inflammatory responses and immune regulation. This systematic review and meta-analysis aimed to clarify the associations between VDBP gene polymorphisms and respiratory diseases including chronic obstructive pulmonary disease(COPD), asthma, Corona Virus Disease-2019(COVID-19) and tuberculosis, so as to provide evidence for the formulation of individualized prevention and therapeutic strategies in the future.

**Methods:**

We searched the Pubmed, web of science, Wanfang, Embase and China National Knowledge Infrastructure(CNKI) to find the eligible case–control studies. Study quality was assessed using the Newcastle–Ottawa Scale (NOS). Forest plots were generated using Review Manager version 5.4, and pooled odds ratios (ORs) with 95% confidence intervals (CIs) were calculated.

**Results:**

A total of 30 studies involving 8,844 participants were included. No significant associations were observed between the rs7041 or rs4588 polymorphism and COPD susceptibility (both *P* > 0.05). In the analysis of GC variants, GC*1F-related genetic models were associated with an increased risk of COPD. The ORs for GC*1F/1F versus GC*1F/1S and for GC*1F/1F versus GC*1S/1S group were 1.73 and 2.17, respectively. The OR for the GC*1F/1F homozygous comparison was 1.96, and the GC*1F allele analysis was also statistically significant (*P* = 0.0003). Ethnicity-based subgroup analyses suggested that GC*1F may be primarily associated with increased COPD susceptibility in East Asian populations. In non-East Asian populations, GC*2 showed a trend toward a reduced risk of COPD, although this association did not reach statistical significance (OR = 0.52, *P* = 0.09). In the asthma analysis, GC*2 may be associated with an increased risk of asthma (*P* ≤ 0.001). However, because relatively few studies were available for asthma, COVID-19, and tuberculosis, and substantial heterogeneity was present in some analyses, the current evidence remains insufficient to draw definitive conclusions regarding the associations between GC variants and these diseases.

**Conclusion:**

The GC*1F allele and GC*1F/1F diplotype may be associated with increased susceptibility to COPD, particularly in East Asian populations, whereas no significant associations were observed between rs7041 or rs4588 and COPD risk. GC*2 may show a potentially protective trend against COPD in non-East Asian populations but may be associated with increased asthma susceptibility. Given the limited and highly heterogeneous evidence regarding asthma, COVID-19, and tuberculosis, further high-quality studies are needed to validate these findings.

**Supplementary Information:**

The online version contains supplementary material available at https://doi.org/10.1186/s12920-026-02443-1.

## Introduction

Respiratory diseases constitute a major global health burden because of high morbidity, disability, and mortality [1, 2]. They include chronic airway diseases, such as chronic obstructive pulmonary disease(COPD) and bronchial asthma, as well as infectious respiratory diseases, including Corona Virus Disease-2019(COVID-19) and tuberculosis. Although these disorders differ substantially in their etiology, clinical manifestations, and pathophysiological mechanisms, their development and progression are influenced by the complex interplay of environmental exposures, host immune status, and genetic background [3, 4]. Single-nucleotide polymorphisms(SNPs) and related genotypes may contribute to disease susceptibility and progression by affecting protein expression, immune regulation, and inflammatory responses [5]. Therefore, identifying susceptibility-related genetic variants and clarifying their roles across different diseases and populations may enhance our understanding of the genetic basis of respiratory disorders and provide a theoretical basis for risk stratification, identification of high-risk individuals, and the development of personalized prevention and management strategies.

Vitamin D-binding protein(VDBP), normally named GC globulin, is a carrier protein that specifically combines vitamin D and its metabolites and transports them to target organs throughout the body. In addition to mediating the transport of vitamin D metabolites, VDBP is involved in the regulation of macrophage function, enhancement of complement C5a-mediated neutrophil chemotaxis, and clearance of extracellular actin released following tissue injury [6, 7].

VDBP is encoded by the GC gene. Two common SNPs, rs7041 and rs4588, located in exon 11 of the GC gene, combine to define the three major isoforms(GC*1F, GC*1S, and GC*2) and six genotypes (homozygotes GC*1F/1F, GC*1S/1S, GC*2/2; heterozygotes GC*1F/1S, GC*2/1F, GC*2/1S) [8, 9] (See Figure S1). Specifically, the GC*1F is characterized by rs7041-T and rs4588-C, GC*1S by rs7041-G and rs4588-C, and GC*2 by rs7041-T and rs4588-A [8] (See Figure S1). These polymorphisms may influence the structure, circulating concentration, and vitamin D-binding properties of VDBP, thereby affecting the transport and bio-availability of vitamin D and its metabolites [9]. Rs7041 may primarily affect the binding characteristics of VDBP for vitamin D metabolites. In contrast, rs4588 has been associated more consistently with differences in circulating VDBP concentrations, which vary among genotypes [10, 11]. Through their combined effects on VDBP concentration and function, rs7041 and rs4588 may modulate circulating total and free vitamin D concentrations and thereby contribute to inter-individual differences in susceptibility to a range of diseases.

Recent years, the polymorphism of VDBP has become an active area of investigation in the area of respiratory genetics. Large body of researches has investigated common genetic variants in GC, the gene encoding VDBP, and their associations with susceptibility to a range of respiratory diseases, including COPD, asthma, COVID-19, and tuberculosis [12, 13]. The variants most frequently examined include rs7041 and rs4588, as well as the GC*1F, GC*1S, and GC*2 haplotypic alleles. However, several meta-analyses only focused on the relationship between VDBP and COPD [7, 14, 15], which did not integrate the main respiratory diseases together. The findings have been inconsistent and heterogeneous, and no clear consensus has yet been researched. Consequently, it remains uncertain whether the polymorphism of VDBP become the independent risk factor, which may prevent their potential application in genetic risk stratification and clinical prediction for respiratory diseases to some extent.

The evidence regarding the associations of rs4588 and the GC*1F, GC*1S, and GC*2 alleles with the aforementioned diseases was systematically evaluated. Given the distinct etiologies and pathophysiological mechanisms of these diseases, disease-stratified analyses were conducted as the primary approach. The effects of different genetic variants were further compared across diseases and populations to determine whether GC polymorphisms exhibit disease-specific or population-specific associations.

## Methods

This study was conducted and reported according to the PRISMA 2020 guidelines.

### Search strategy

We systematically searched PubMed, Web of Science, Embase, Chinese National Knowledge Infrastructure (CNKI), and WanFang databases for research on the relationship between VDBP genetic polymorphisms and the risk of respiratory disease from January 1990 to March 2026. The search terms used were as follows: ["vitamin D binding protein" or "GC globulin" or "VDBP"] and ["respiratory system disease" or "chronic airway disease" or "chronic obstructive pulmonary disease" or "COPD" or "chronic bronchitis" or "emphysema" or "asthma" or "bronchial asthma" or "bronchiectasis" or "COVID-19" or "Corona Virus Disease 2019" or "TB" or "tuberculosis"].(Supplementary Table 4) The literature language was restricted to English and Chinese, and subjects were limited to humans. References of these selected papers were also manually screened to identify potentially relevant studies.

### Inclusion and exclusion criteria

The inclusion criteria were as follows: (1) research on the association between VDBP gene polymorphisms and respiratory diseases, (2) case–control studies in English or Chinese, (3) the control group comprising healthy individuals,who were excluded from the mentioned respiratory diseases on the basis of history and symptom, For COVID-19 studies, we explicitly distinguished between susceptibility, severity, mortality, and other clinical phenotypes. These studies were organized by outcome type for analysis, and meta-analysis was conducted only when at least three studies addressed the same outcome. (4) provide the frequency data of each genotype in the case group and control group that can be calculated, (5) sufficient data were provided for estimating odds ratios (OR) and 95% confidence interval (*95%CI*).

The exclusion criteria were: (1) reviews, meta-analyses, case reports, letters, and editorials; (2) repetitive study; (3) studies with control groups deviating from Hardy–Weinberg equilibrium (HWE; *P* < 0.05) were excluded.

### Data extraction

Two researchers independently extracted data from eligible studies and resolved disputes through discussion. For each study, we recorded the first author, year of publication, country of study, number of cases and controls, average age and smoking history (including years) of cases and controls, gender distribution, forced expiratory volume in one second (FEV_1_), percentage predicted values for FEV_1_ (FEV_1_%), forced expiratory volume in one second/forced vital capacity (FEV_1_/FVC), allele and genotype frequency of case group and control group (Table 1).

**Table 1:** The characterize of the COPD study

	Study	Country	Subjects (n)		Age (year)		Sex (n) M/F		Smoking (pack-year)		FEV_1_ (L)		FEV_1_% (%)		FEV_1_/FVC (%)	
			Case	Control	Case	Control	Case	Control	Case	Control	Case	Control	Case	Control	Control	Case
COPD	Al-Azzawi 2017	Egyptian	80	80	54.98 ± 9.03	53.05 ± 7.40	58/18	62/18	46.4 ± 1.7	33.7 ± 1.2					59.0 ± 3.7	
	Chuaychoo 2018	Thailand	136	68	71.04 ± 8.84	65.45 ± 8.96			46.50 ± 54.12	1.73 ± 3.85	1.31 ± 0.52	2.61 ± 0.50	63.88 ± 24.60	111.46 ± 18.01	51.22 ± 12.28	78.93 ± 11.36
	Gao 2020	Finland	44	189			38/91	98/91								
	Horne 1990	Canada	104	413												
	Huang 2007	China	75	69	60 ± 6	60 ± 6	75/0	69/0								
	Ishii 2001	Japan	63	82												
	Ishii 2014	Japan	361	219	68.9 ± 8.5	63.1 ± 12.0	331/37	182/37			1.7 ± 0.7	2.8 ± 0.8	60.3 ± 20.4	95.8 ± 15.5	51.6 ± 11.6	78.6 ± 5.8
	Ito 2004	Japan	103	88	67.4 ± 7.8	60.8 ± 12.0	99/16	72/16	58.3 ± 29.1	41.1 ± 22.2	1.18 ± 0.49	2.66 ± 0.86	45.3 ± 18.4	88.4 ± 20.7	45.8 ± 10.4	80.1 ± 7.8
	Janssens 2010	Belgium	262	152	66 (60, 72)	61 (58, 65)	215/32	120/32	47 (33, 63)	39 (30, 52)	1.8 ± 0.9	3.2 ± 0.8	61 ± 27	104 ± 15		
	Jung 2014	Korean	203	157	67 (62, 71)	53 (47, 59)	199/9	148/9	46.0 ± 23.4	30.7 ± 17.4	1.42 ± 0.49	3.17 ± 0.59	53.1 ± 16.9	93.9 ± 12.8	47.5 ± 10.9	78.3 ± 5.1
	Laufs 2004	Iceland	102	183	71.1	42.9	42/78	105/78	38	0						
	Li 2014	China	116	134	68 (61, 74)	58 (51, 56)	113/1	133/1	43 (30, 58)	35 (26, 47)	1.49 ± 0.65	2.58 ± 0.67	74.29 ± 28.66	117.75 ± 20.55		
	Lu 2004	China	69	52	67.1 ± 7.4	64.9 ± 8.6	69/0	52/0								
	Shen 2010	China	100	100	62.3 ± 9.7	60.9 ± 8.6	72/34	66/34	123.5 ± 29.7	25.6 ± 13.3	1.28 ± 0.43	3.57 ± 0.65			43.5 ± 12.8	92.8 ± 5.8
	Wood 2011	UK	93	351												
	Zhou 2017	China	120	107	65.1 ± 8.7	62.8 ± 10.2	116/2	105/2			1.82 ± 0.51	2.73 ± 0.37	52.0 ± 12.6	90.0 ± 5.7		
Asthma	Al-Arifa 2016	Pakistan	200	212			86/145	126/86								
	Fawzy 2019	Egypt	96	96	9.7 ± 3.0	9.6 ± 3.1	44/52	40/56								
	Lahmar 2018	Tunisia	154	154	45.57 ± 13.08	46.36 ± 10.03	26/128	66/88								
	Li 2011	China	467	288	40.8 ± 14.1	45.1 ± 0.7	185/282	158/130								
	Nasiri-Kalmarzi 2020	Iran	110	110	28.73 ± 6.89	31.34 ± 7.81	41/69	49/61								
	Bastyte 2023	Lithuania	63	32	46 ± 2	30 ± 2	17/46	13/19								
	Ashok 2023	South India	207	200	4.5 ± 2.5	7.5 ± 2.5	132/75	118/82								
COVID-19	Hamed 2024	Egypt	140	80	55.5 ± 29.5	62.5 ± 19.5	78/62	54/26								
	Ozturk 2025	Turkey	120	113	64.73 ± 15.32	57.55 ± 16.36	74/46	38/75								
	Santamera 2023	Kazakhstan	134	46	41.49 ± 13.09	43.09 ± 14.01	47/87	20/26								
Tuberculosis	Zhang 2025	China	490	489	44.92 ± 17.77	43.43 ± 12.95	318/172	224/265								
	Lee 2015	Taiwan	198	170	55.78 ± 10.99	55.76 ± 18.62	137/61	79/91								

FEV_1_: Forced expiratory volume in 1 s, FEV_1_%: percentage predicted values for FEV_1_, FVC: Forced vital capacity

### Quality score assessment

Two researchers independently assessed the quality of the included study using the Newcastle Ottawa Scale (NOS). This scale assesses the studies on selection, comparability, and exposure. The scores ranged from 0 to 9, and studies were considered high quality if the score was ≥ 7. When there is a disagreement between the two researchers, they are resolved through discussion. All studies in this research were evaluated as the high quality.

### Statistical analysis

Two groups of allele and genotype frequency data were extracted to evaluate the relationship between SNPs and susceptibility to chronic airway disease.VDBP gene has three different alleles in humans, namely GC*1F, GC*1S and GC*2. They can be combined into six genotypes (GC*1F/1F, GC*1F/1S, GC*1F/2, GC*1S/1S, GC*1S/2, and GC*2/2). Therefore, after the traditional SNP polymorphism analysis, three distinct groups were established for investigation: GC*1F/1S (comprising GC*1F/1F, GC*1F/1S, GC*1S/1S), GC*1F/2 (including GC*1F/1F, GC*1F/2, GC*2/2) and GC*1S/2 (encompassing GC*1S/1S, GC*1S/2, GC*2/2). The meta-analysis and systematic review was conducted using dominant, recessive, and co-dominant genetic models, and one of the models was selected for the final meta-analysis [15]. Additionally, We examined the comparative analysis of individual alleles(GC*1F vs. GC*1S; GC*1F vs. GC*2; GC*1S vs. GC*2). Based on the genotype frequency in cases and controls, an odds ratio (OR) with a 95% confidence interval (*95%CI*) was used to assess the strength of the association between the VDBP gene polymorphism and the risk of COPD and asthma. We used *I*^*2*^ statistics to evaluate heterogeneity. The combined OR was calculated using fixed (*I*^*2*^ < 50) or random (*I*^*2*^ ≥ 50) effect models. The Mantel–Haenszel (M-H) method was used for the meta-analysis of binary variables. To identify potential sources of heterogeneity, subgroup analyses were conducted based on ethnicity distinguish between East Asian and non-East Asian population(including European, South Asian and Middle-Eastern). Meta-analyses were performed using Review Manager 5.4 software. Egger’s tests were performed using Stata to assess publication bias only for comparisons including ≥ 10 studies.

Due to the exploratory nature of several analyses and the limited number of available studies for certain diseases, a formal correction for multiple testing was not implemented. Therefore, nominal *P* values are presented, and the findings should be interpreted with the consideration of the number of comparisons performed.

Associations were assessed under five genetic (models-allelic, dominant, recessive, homozygous, and heterozygous), which were predetermined. The method proposed by Thakkinstian et al. [15] was used to determine the genetic model most consistent with the observed genotype-specific odds ratios.

### Patient and public involvement

No patients participated in the design, conduct, reporting, or dissemination of this meta-analysis. The research question and outcomes were determined based on existing clinical guidelines and the published literature. The findings will be shared via open-access publication and conference presentations.

## Results

### Study characteristics

**Fig. 1:**
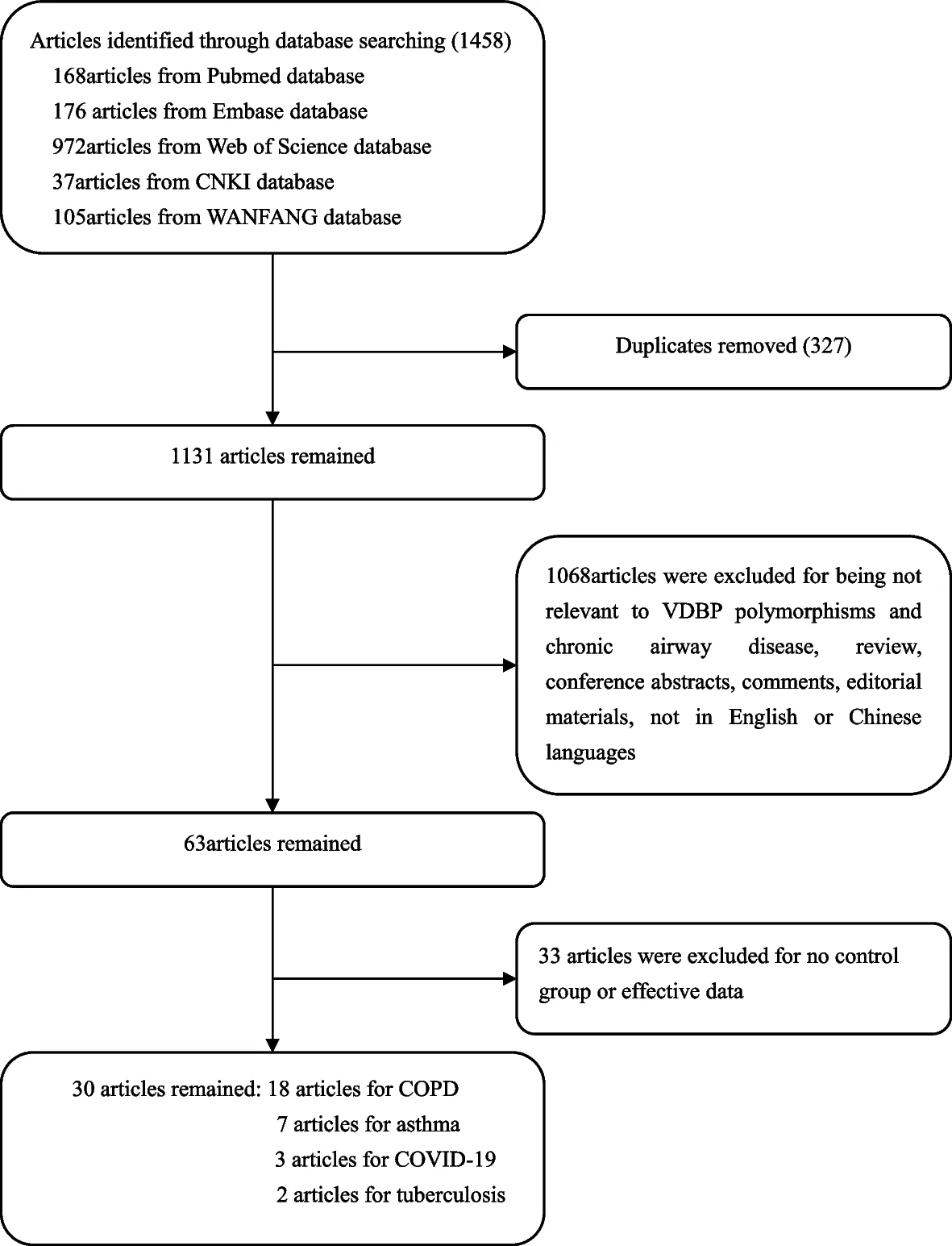
Flow diagram for the selection of qualified studies

The initial literature search identified 1,458 studies. After eliminating 327 duplicate records, 1,068 studies were excluded for various reasons, and 33 were excluded for the lack of control groups or insufficient data. Ultimately, thirty articles were included in the final analysis: eighteen for COPD [16–33], seven for asthma [12, 34–39], three articles for COVID-19 [40–42] and two for tuberculosis [43, 44]. See Fig. 1.

Regarding the association between COPD and VDBP gene polymorphisms, a total of 4,475 subjects from 10 countries were enrolled in this study spanning the years 1990 to 2020. To investigate the association between asthma and VDBP gene polymorphisms, seven studies were included, involving a total of 2,389 asthmatic patients recruited from 2011 to 2025. Regarding COVID-19, 633 participants were enrolled in the study from 2023 to 2025. Additionally, 1,347 clinical samples collected in this study from 2015 to 2025 were analyzed to examine the association between tuberculosis susceptibility and VDBP gene polymorphisms. The most commonly employed genotyping method across these studies was PCR–RFLP, as detailed in Table 1. The equality of cohort studies was evaluated using the Newcastle–Ottawa Scale (NOS) tool, and the quality assessment of all eligible articles is presented in Table 2.

**Table 2:** Quality assessment of studies included in the meta-analysis

	Study	Selection				Comparability		Exposure			Total scores
		1	2	3	4	1	2	1	2	3	
	Al-Azzawi 2017	☆	☆		☆	☆		☆	☆	☆	7
COPD	Chuaychoo 2018	☆	☆	☆	☆	☆		☆	☆	☆	8
	Gao 2020	☆	☆		☆	☆		☆	☆	☆	7
	Horne 1990	☆	☆		☆	☆		☆	☆	☆	7
	Huang 2007	☆	☆		☆	☆		☆	☆	☆	7
	Ishii 2001	☆	☆	☆	☆	☆		☆	☆	☆	7
	Ishii 2014	☆	☆		☆	☆		☆	☆	☆	7
	Ito 2004	☆	☆	☆	☆	☆		☆	☆	☆	8
	Janssens 2010	☆	☆	☆	☆	☆		☆	☆	☆	8
	Jung 2014	☆	☆	☆	☆	☆		☆	☆	☆	8
	Laufs 2004	☆	☆		☆	☆		☆	☆	☆	7
	Li 2014	☆	☆	☆	☆	☆	☆	☆	☆	☆	9
	Lu 2004	☆	☆		☆	☆		☆	☆	☆	7
	Shen 2010	☆	☆		☆	☆		☆	☆	☆	7
	Wood 2011	☆	☆		☆	☆		☆	☆	☆	7
	Zhou 2017	☆	☆		☆	☆		☆	☆	☆	7
Asthma	Al-Arifa 2016	☆	☆	☆	☆	☆		☆	☆	☆	8
	Fawzy 2018	☆	☆		☆	☆		☆	☆	☆	7
	Lahmar 2018	☆	☆		☆	☆		☆	☆	☆	7
	Li 2011	☆	☆		☆	☆		☆	☆	☆	7
	Nasiri-Kalmarzi 2020	☆	☆		☆	☆		☆	☆	☆	7

Selection: 1, is the case definition adequate? (if some independent validation was required, one star); 2, representativeness of the cases (if yes, one star); 3, selection of controls (if they were from community controls, one star); 4, definition of controls (if they had no history and new occurrence, one star). Comparability: comparability of cases and controls on the basis of design or analysis: 1, ethnicity (if yes, one star); 2, age (if yes, one star). Exposure: 1, ascertainment of exposure (if in reliable method, one star); 2, same method of ascertainment for cases and controls (if yes, one star); 3, non-response rate (if they were the same between cases and controls, one star). COPD: chronic obstructive pulmonary disease

### COPD and VDBP gene polymorphisms

#### rs7041/rs4588 meta-analysis

**Fig. 2:**
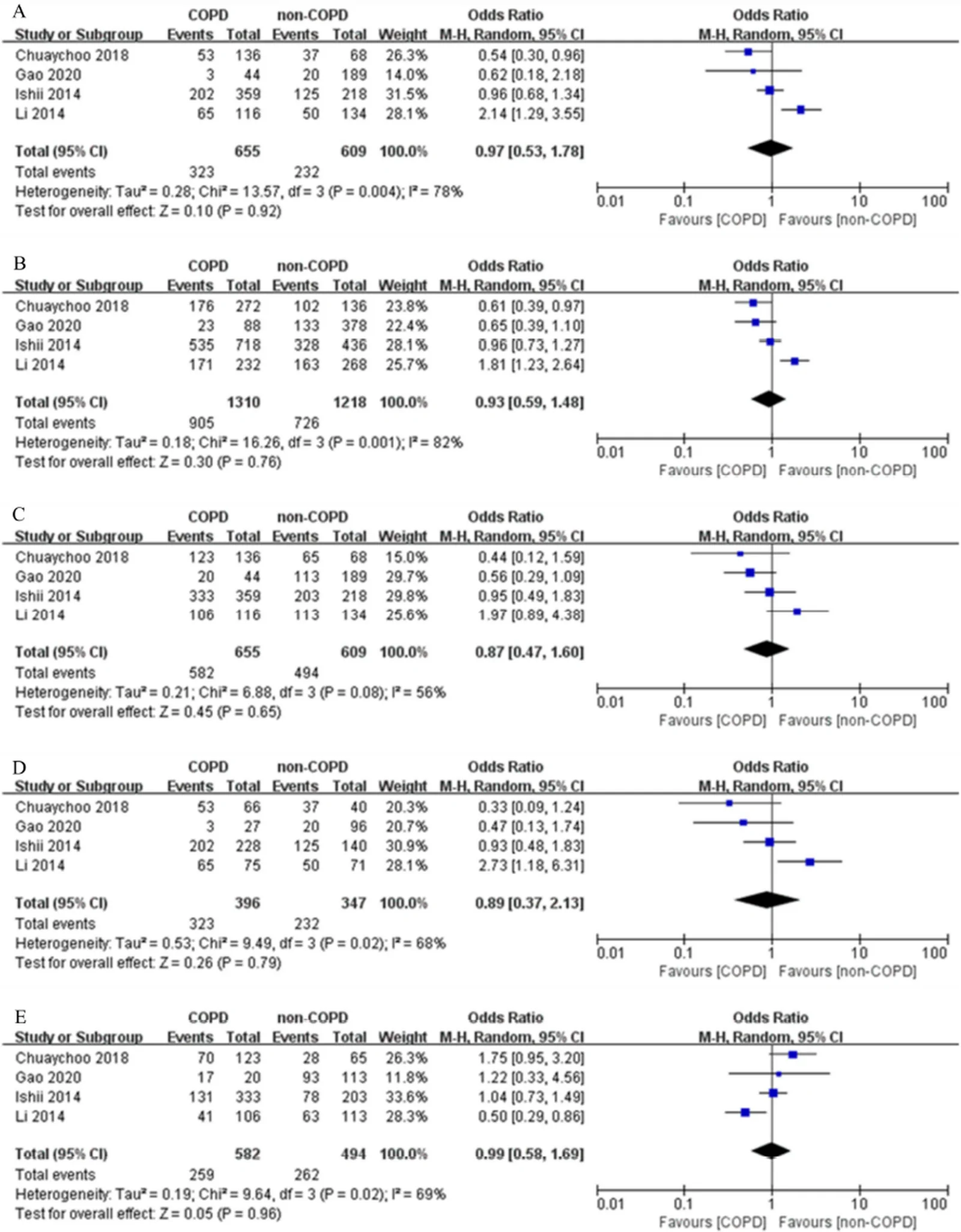
Forest plots of VDBP gene rs7041 polymorphism with COPD under five inheritance models. **A** Allele model, **B** Dominant model, **C** Recessive model, **D** Homozygous model, **E** Heterozygous model. VDBP: vitamin D binding protein, COPD: chronic obstructive pulmonary disease

Firstly, we analyzed the association of rs7041 polymorphism (G as the alternative allele) with COPD risk across five genetic analyses. Due to significant heterogeneity test *I*^*2*^ > 50%, random effect model was used for analysis. Our study found that there was no significant difference in the allele model (G vs. T), the dominant gene model (TG + GG vs. TT), the recessive gene model (TT + TG vs. GG), the homozygous model (GG vs. TT), and the heterozygous model (TG vs. TT) (*P* > 0.05). See Fig. 2.

**Fig. 3:**
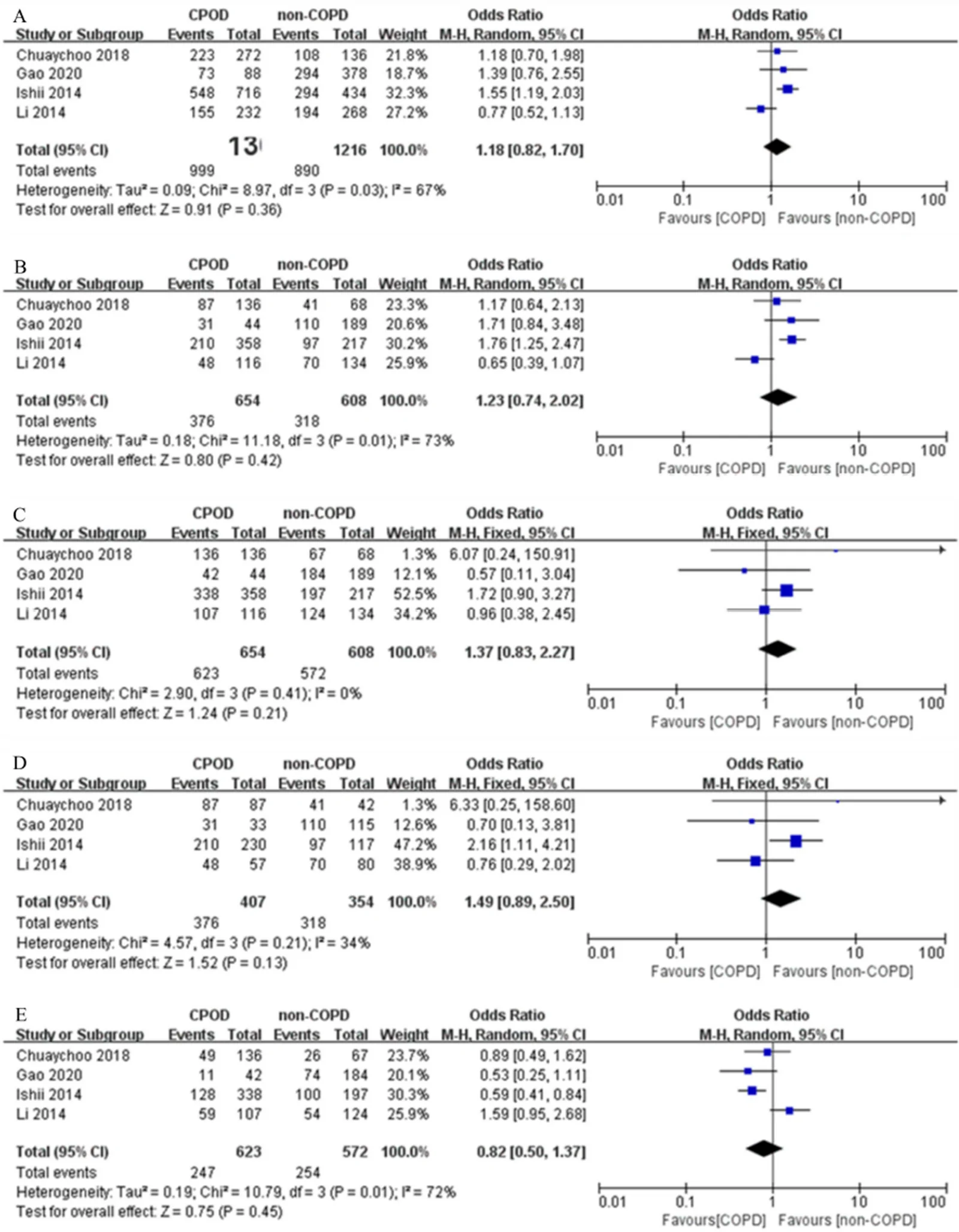
Forest plots of VDBP gene rs4588 polymorphism with COPD under five inheritance models. **A** Allele model, **B** Dominant model, **C** Recessive model, **D** Homozygous model, **E** Heterozygous model. VDBP: vitamin D binding protein. COPD: chronic obstructive pulmonary disease

Similarly four studies were included to assess the association between the SNP and COPD susceptibility, with A defined as the alternative allele [17, 18, 21, 28]. No significant differences were observed in any of the tested genetic models, including the allelic model (A vs. C), the dominant gene model (CA + AA vs. CC), the recessive gene model (CC + CA vs. AA), the heterozygous model (CA vs. CC), and the homozygous model (AA vs. CC) (*P* > 0.05). See Fig. 3.

#### The single allele (GC* 1 F or GC*1S or GC*2) comparison

**Fig. 4:**
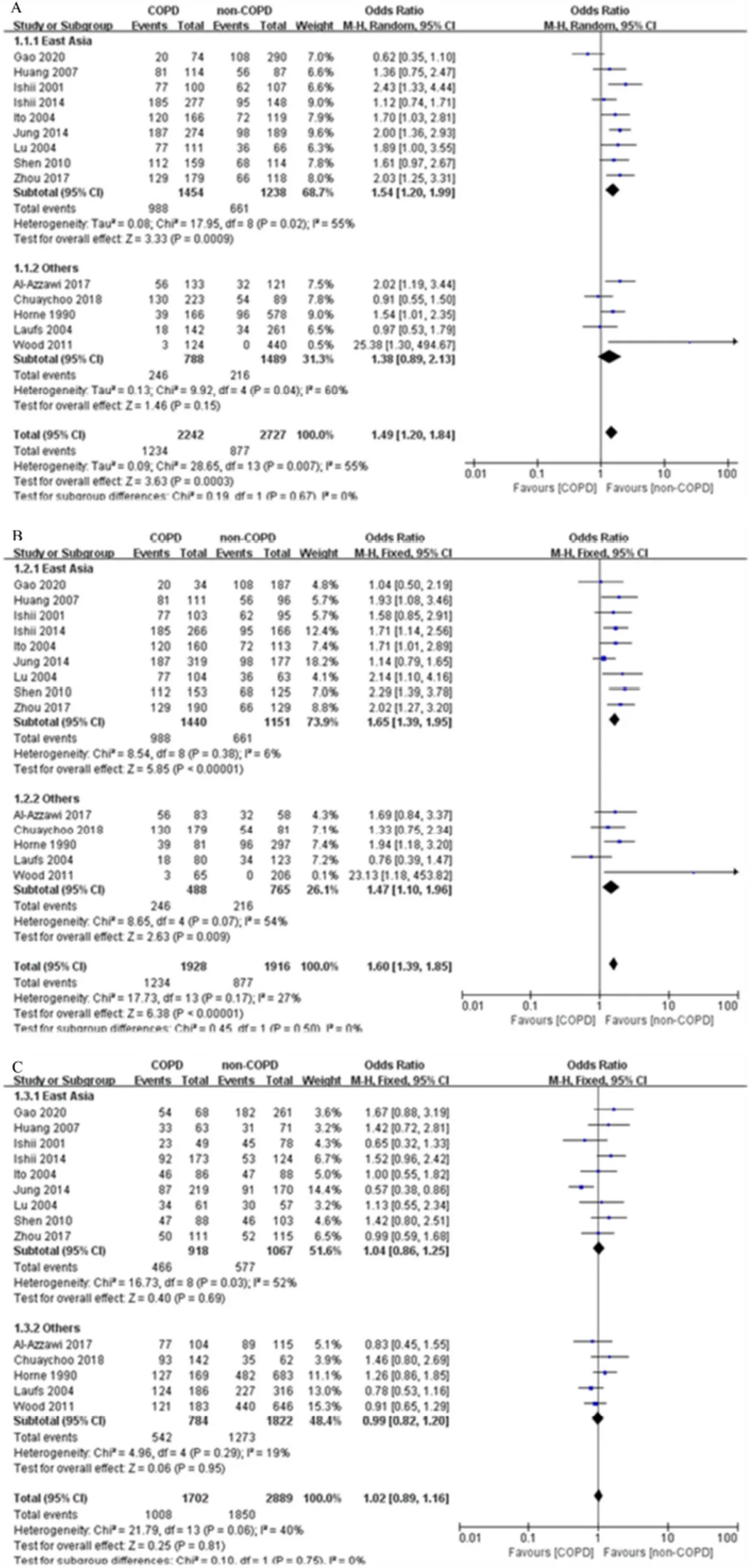
Forest plots of the association between the VDBP single allele and risk of COPD. **A** GC*1F vs. GC*1S, **B** GC*1S vs. GC*2, **C** GC*1F vs. GC*2. VDBP: vitamin D binding protein. COPD: chronic obstructive pulmonary disease

To evaluate the associations of different VDBP isoforms with the risk of COPD, a meta-analysis was conducted incorporating data from 14 studies [18, 20–22, 24, 26, 29, 31, 33].The results showed that the presence of the GC*1F allele was associated with a 1.49-fold increased risk compared to GC*1S allele (OR 1.49, *95%CI* 1.20–1.84, *P* = 0.0003, *I*^*2*^ = 55%) and a 1.60-fold increased risk compared to GC*2 allele (OR 1.60, *95%CI* 1.39–1.85, *P* < 0.001, *I*^*2*^ = 27%)(See Fig. 4A and C). No statistically significant difference was detected between the GC*1S and GC*2 alleles (OR 1.02, *95%CI* 0.89–1.16, *P* = 0.81, *I*^*2*^ = 40%) (Fig. 4B). Additionally, the analysis did not reveal any significant publication bias when comparing the three alleles (Egger's test, *P* = 0.133, 0.525, 0.183, respectively).

Subgroup analysis demonstrated that the results for East Asians populations were consistent with those of the overall population. However, no statistically significant difference was observed between the GC*1F and GC*1S alleles in non-East Asians (OR 1.38, *95%CI* 0.89–2.13, *P* = 0.15).

#### The most appropriate genetic models

**Fig. 5:**
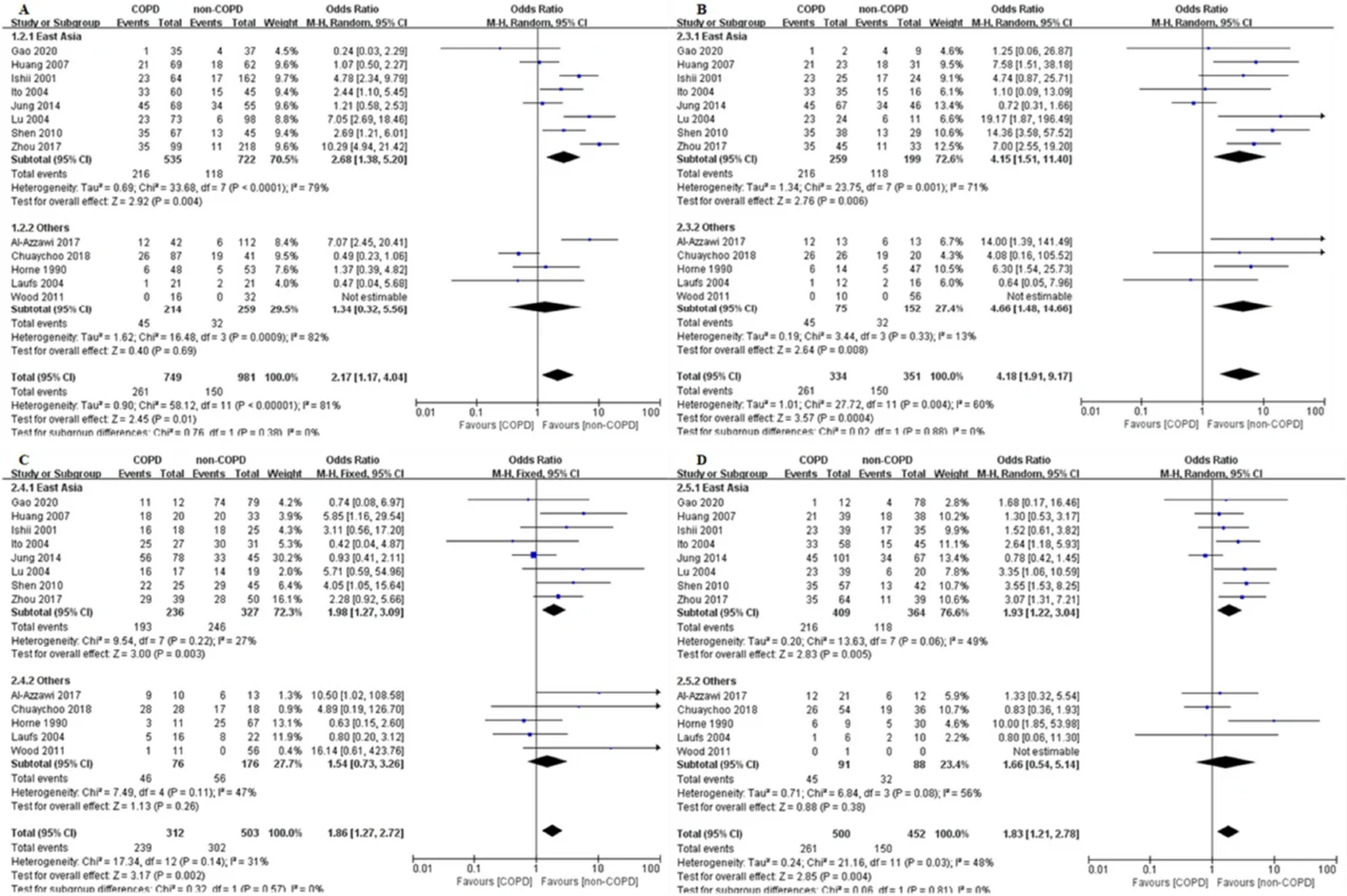
Forest plots of the VDBP gene polymorphisms and the risk of COPD in the appreciated genetic model. **A** Recessive model (GC*1F/1F vs. GC*1F/1S + GC*1S/1S), **B** Co-dominant model (GC*1F/1F vs. GC*2/2), **C** Co-dominant model (GC*1F/2 vs. GC*2/2), **D** Co-dominant model (GC*1F/1F vs. GC*1F/2)

Dominant, recessive, and co-dominant genetic models were constructed for the three alleles groups(GC*1F/1S, GC*1F/2, and GC*1S/2, see Table 3) [15]. In the GC*1F/1S model, the OR with *95%CI* for the co-dominant model comparisons of GC*1F/1F vs. GC*1S/1S and GC*1F/1F vs. GC*1F/1S were 2.17 (1.45–3.25) and 1.73 (1.15–2.60), respectively, both with *P* < 0.05. Consequently, the recessive model (GC*1F/1F vs. GC*1F/1S + GC*1S/1S) was selected for further analysis. The risk of COPD associated with the 1F/1F genotype was found to be 2.17 times higher than that associated with the GC*1F/1S + GC*1S/1S genotype (OR 2.17, *95%CI* 1.17–4.04, *P* = 0.01, *I*^*2*^ = 81%). The heterogeneity may result from the differences in vitamin D affinity and plasma concentrations among these subtypes. Subgroup analysis revealed that this association remained significant in East Asian populations, whereas no statistically significant association was observed in non-East Asian populations(OR 2.68, *95%CI* 1.38–5.20, *P* = 0.004) (Fig. 5A).

**Table 3:** Overall data analysis in the dominant, recessive, and co-dominant models

	Models	*P*	OR (95%CI)	*I*^*2*^ (%)	*P* for heterogeneity	*P* for Egger’s test
GC 1F/1S	Dominant (1F-1F + 1F-1S vs. 1S-1S)	0.001	2.14 (1.36–3.36)	61	0.002	
	Recessive (1F-1F vs. 1F-1S + 1S-1S)	0.010	2.17 (1.17–4.04)	81	< 0.001	0.518
	Co-dominant (1F-1F vs. 1S-1S)	< 0.001	2.17 (1.45–3.25)	0	0.480	
	Co-dominant (1F-1S vs. 1S-1S)	0.150	1.24 (0.92–1.66)	0	0.480	
	Co-dominant (1F-1F vs. 1F-1S)	0.008	1.73 (1.15–2.60)	49	0.030	
GC1F/2	Dominant (1F-1F + 1F-2 vs. 2–2)	0.002	2.61 (1.43–4.76)	50	0.020	
	Recessive (1F-1F vs. 1F-2 + 2–2)	< 0.001	2.23 (1.41–3.53)	62	0.002	
	Co-dominant (1F-1F vs. 2–2)	< 0.001	4.18 (1.91–9.17)	60	0.004	0.263
	Co-dominant (1F-2 vs. 2–2)	0.002	1.86 (1.27–2.72)	31	0.140	0.069
	Co-dominant (1F-1F vs. 1F-2)	0.004	1.83 (1.21–2.78)	48	0.030	0.386
GC1S/2	Dominant (2–2 + 1S-2 vs. 1S-1S)	0.430	0.91 (0.72–1.15)	0	0.580	1.360
	Recessive (2–2 vs. 1S-2 + 1S-1S)	0.060	0.61 (0.37–1.03)	49	0.020	0.078
	Co-dominant (2–2 vs. 1S-1S)	0.120	0.75 (0.53–1.07)	40	0.070	0.536
	Co-dominant (1S-2 vs. 1S-1S)	0.990	1.00 (0.77–1.29)	0	0.570	0.435
	Co-dominant (2–2 vs. 1S-2)	0.050	0.58 (0.33–1.00)	49	0.020	0.048

In the GC*1F/2 model, the OR with *95%CI* for the co-dominant model comparisons of GC*1F/1F vs. GC*2/2,(Fig. 5B) co-dominant model (GC*1F/2 vs. GC*2/2 (Fig. 5C), and GC*1F/1F vs. GC*1F/2 (Fig. 5D) were 4.18 (1.91–9.17), 1.86 (1.27–2.72) and 1.83 (1.21–2.78) respectively (*P* < 0.05). Therefore, the co-dominant model (GC*1F/1F vs. GC*1F/2 vs. GC*2/2) was employed for analysis. Subgroup analysis indicated a similar significant difference in East Asians but not in non-East Asians (Fig. 5B).

In the GC*1S/2 model, the co-dominant model comparisons (GC*2/2 vs. GC*1S/1S, GC*1S/2 vs. GC*1S/1S and GC*2/2 vs. GC*1S/2) did not show a significant difference with COPD (*P* > 0.05). Therefore, three genetic models were evaluated: the dominant model(GC*2/2 + GC*1S/2 vs. GC*1S/1S); the recessive model(GC*2/2 vs. GC*1S/2 + GC*1S/1S), and the co-dominant model(GC*2/2 vs. GC*1S/2 vs. GC*1S/1S). No significant associations were identified in any of models. Subgroup analyses also failed to demonstrate significant associations.

#### Analysis of homozygous genes (GC*1F/1F or GC*1S/1S or GC*2/2) vs. other genotypes

**Fig. 6:**
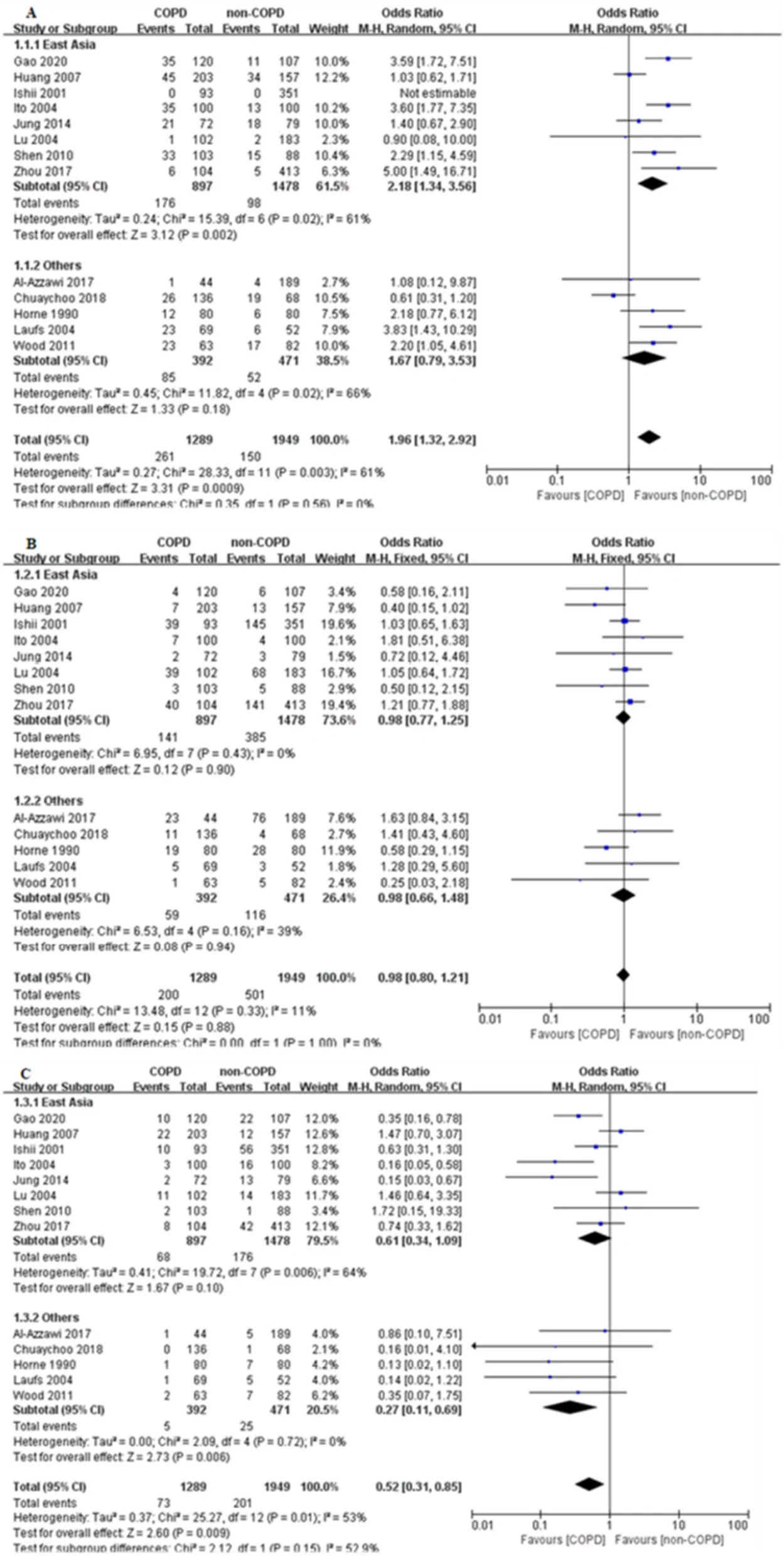
Forest plots of the association between the VDBP homozygous genes vs. other genotypes and the risk of COPD (analysis of homozygous genes vs. other genotypes). **A** GC*1F/1F vs. GC*1F/1S + GC*1S/1S + GC*2/1S + GC*2/1F + GC*2/2, **B** GC*1S/1S vs. GC*1F/1F + GC*1F/1S + GC*2/1S + GC*2/1F + GC*2/2, **C** GC*2/2 vs. GC*1F/1F + GC*1F/1S + GC*1S/1S + GC*2/1S + GC*2/1F. VDBP: vitamin D binding protein, COPD: chronic obstructive pulmonary disease

Our study determined that individuals with the GC*1F/1F genotype had a 1.96-fold increased risk of developing COPD compared to those with other genotypes (OR 1.96, *95%CI* 1.32–2.92, *P* = 0.0009). However,moderate heterogeneity was observed among the included studies (*I*^*2*^ = 61%), suggesting potential variability in study characteristics and populations. Subgroup analysis demonstrated a significant association in East Asians (Fig. 6A), where the risk of COPD was 2.18 times higher compared to other genotypes (OR 2.18, *95%CI* 1.34–3.56, *P* = 0.002, *I*^*2*^ = 61%), but this was not observed in non-East Asians.This finding implies that ethnic background and gene-environment interactions may contribute to these results.

Conversely, the GC*2/2 genotype exhibited a protective effect against COPD, with a risk reduction of 48% compared to other genotypes (OR 0.52, *95%CI* 0.31–0.85, *P* = 0.009, *I*^*2*^ = 53%). The subgroup analysis revealed that the protective effect was not statistically significant among East Asians (*P* = 0.10), where it was notably significant in non-East Asian populations (OR 0.27, *95%CI* 0.11–0.69, *P* = 0.006, *I*^*2*^ = 0%). The overall heterogeneity observed was primarily attributed to this ethnic (Fig. 6B).

No significant differences were found between the GC*1S/1S genotype and other genotypes (OR 0.98, *95%CI* 0.80–1.21, *P* = 0.88, *I*^*2*^ = 11%) and the subgroup analysis also showed no significant difference (Fig. 6C). Additionally,no significant publication bias was detected for comparisons of homozygous genotypes(GC*1F/1F; GC*2/2; GC*1S/1S) versus other genotypes (Egger's test, *P* = 0.281, 0.294, 0.063, respectively).

### Asthma and VDBP gene polymorphisms

#### rs7041/rs4588 meta-analysis and systematic review

**Fig. 7:**
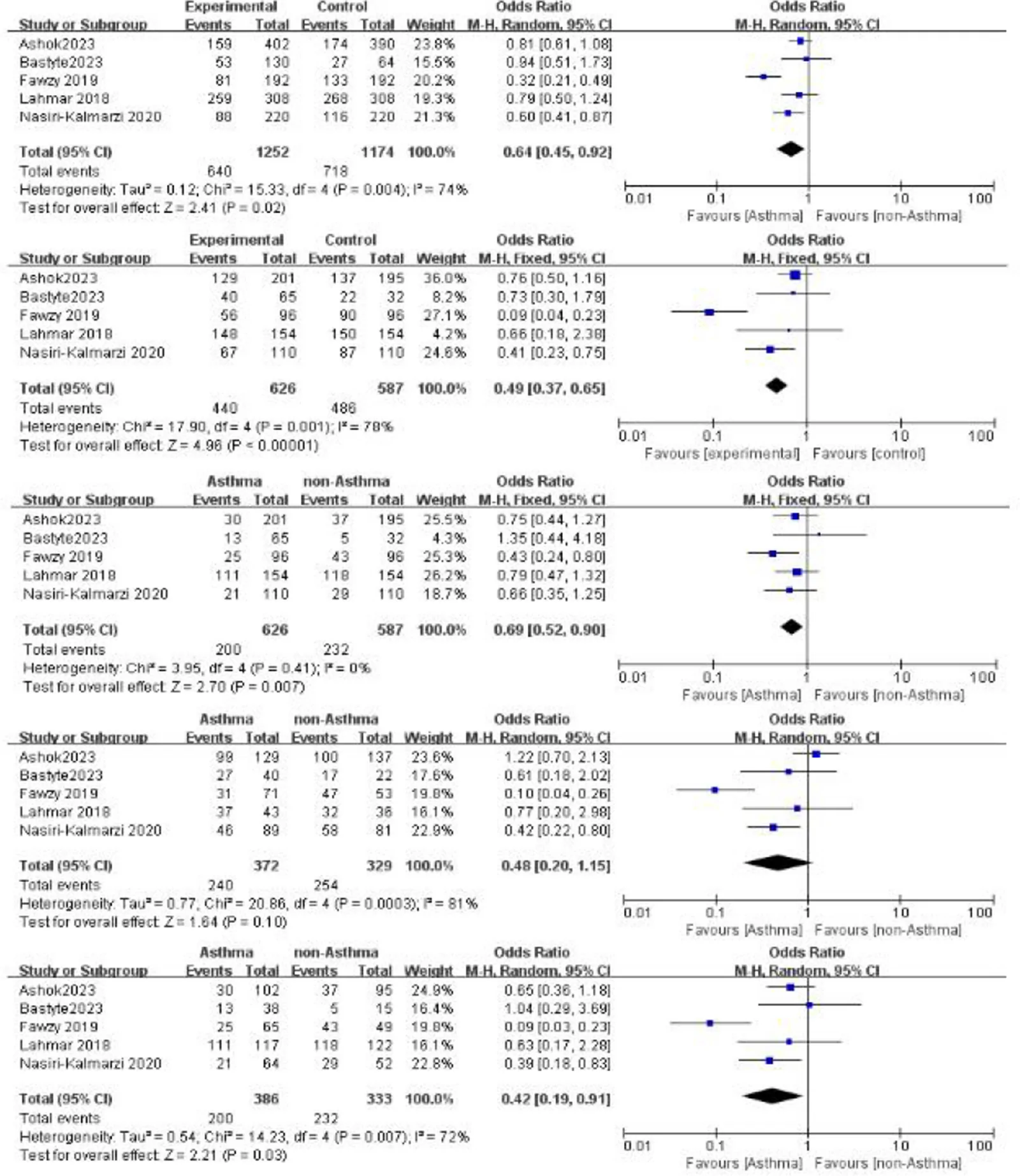
Forest plots of VDBP gene rs7041 polymorphism with asthma under five inheritance models. **A** Allele model, **B** Dominant model, **C** Recessive model, **D** Homozygous model, **E** Heterozygous model. VDBP: vitamin D binding protein

The association between the rs7041 polymorphism of the VDBP and asthma was evaluated under five genetic models: allelic model (G vs. T), dominant model (TG + GG vs. TT), recessive model (TT + TG vs. GG), heterozygous model (TG vs. TT), homozygous model (GG vs. TT). Notably, a significant association was absent only in the heterozygous model (*P* = 0.10). See Fig. 7.

Only two studies have examined the association between rs4588 gene polymorphism and asthma. Fawzy et al. suggested that the rs4588 AA genotype and A allele provided a protective effect against asthma [35]. Lahmar et al. also suggested that rs4588 was associated with reduced risk of developing atopic asthma (OR 0.36, 95% CI 0.13–0.96, *P* = 0.04) [37].

#### A systematic review of the single allele (GC* 1 F or GC*1S, or GC*2) comparison

Al-Arifa et al. [36] found that homozygous GC*2 was significantly associated with increased asthma risk (*P* ≤ 0.001), while homozygous GC*1S (*P* = 0.250) was less common in asthma patients. Similarly, Li et al. [38] reported that GC*2 was significantly associated with asthma risk when compared to GC*1S/1F genotypes (OR = 1.35, *95%CI* 1.01–1.78, *P* = 0.006). The high rate of GC*1S genotype in the control group may suggest a protective effect against asthma susceptibility.

The contrasting associations of GC*2 and GC*1S with asthma susceptibility suggest that VDBP variants may have value as candidate markers for future genetic risk stratification and may help identify individuals who warrant closer monitoring.

#### Sensitivity analysis

Sensitivity analysis demonstrated that no single study significantly influenced the pooled estimates, indicating the stability of the results.

### COVID-19 and VDBP gene polymorphisms

#### rs7041/rs4588 meta-analysis and systematic review

Three studies were included for COVID-19, each addressing different clinical outcomes such as susceptibility, severity, mortality, or specific phenotypes. Due to the heterogeneity of outcome definitions, no pooled meta-analysis was performed across all studies. Instead, the findings are presented below stratified by outcome type, followed by a cautious genetic model analysis of the rs7041 polymorphism.

One study by Sarría-Santamera et al. [45] focused on asymptomatic cases and reported that the recessive model of rs7041 (TT + TG vs. GG) was associated with a decreased susceptibility to COVID-19 (OR = 0.37, *95%CI* 0.15–0.93, *P* = 0.034). Hamed et al. [41] examined patients with different clinical outcomes and found that the GT genotype of rs7041 and the AC genotype of rs4588 were positively correlated with COVID-19 mortality, whereas the TT genotype was negatively correlated with disease prevalence. These findings align with those of Öztürk et al. [42], who reported a significant association between the rs7041 polymorphism and pancreatic involvement in COVID-19 patients (*P* = 0.004).

**Fig. 8:**
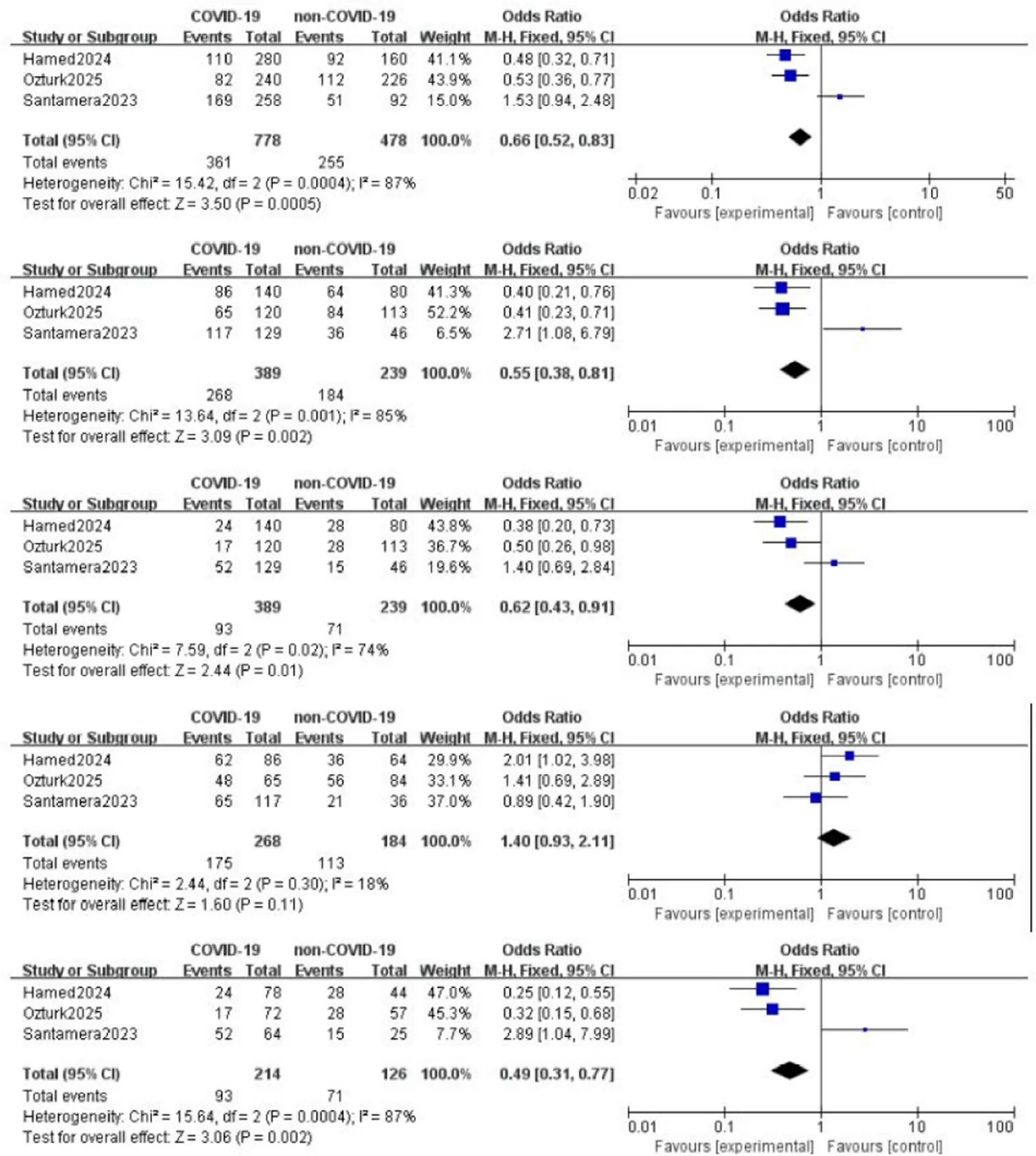
Forest plots of VDBP gene rs7041 polymorphism with COVID-19 under five inheritance models. **A** Allele model, **B** Dominant model, **C** Recessive model, **D** Homozygous model, **E** Heterozygous model. VDBP: vitamin D binding protein

Despite the heterogeneity of outcome definitions, a meta-analysis of the rs7041 polymorphism was conducted across the three studies under five genetic models for exploratory purposes, acknowledging that the results should be interpreted with caution. As illustrated in Fig. 8, no significant association was identified in the heterozygous model (TG vs. TT: *P* = 0.30, *I*^*2*^ = 18%). In contrast, statistically significant associations were observed in the allelic model (G vs. T), the dominant model (TG + GG vs. TT), the recessive model (TT + TG vs. GG), and the homozygous model (GG vs. TT), with all uncorrected *P* < 0.05. Nevertheless, due to the limited number of studies and the combination of susceptibility, severity, and mortality outcomes, these pooled estimates should be regarded as exploratory and necessitate validation in larger, more homogeneous cohorts.

### Tuberculosis and VDBP gene polymorphisms

#### A systematic review of the rs7041/rs4588 comparison

Only two studies were available concerning tuberculosis, which is insufficient for a reliable meta-analysis. Consequently, we provide a systematic review of the individual study findings rather than pooled estimates. Zhang et al. [44, 46] found no significant association between the dominant genetic model of rs7041 and tuberculosis susceptibility (*P* > 0.05), whereas the rs4588 TG genotype may play a role in the pathogenesis of TB (OR = 0.687, *95%CI* 0.527–0.896,* P* = 0.006). Conversely, Lee et al. [45] reported that carriers of the rs7041 A allele (AA + AC) were at an increased risk of TB susceptibility (OR = 2.24, *95%CI* 1.04–4.80, *P* = 0.035).

Due to the conflicting results and the limited number of studies, a pooled estimate was not calculated,leaving the relationship between VDBP polymorphisms and tuberculosis inconclusive. Additional large-scale studies are needed to clarify this association.

## Discussion

This meta-analysis and systematic review is designed to comprehensively examine the associations between common VDBP gene polymorphisms and the risk of multiple respiratory diseases. Relative research has verified VDBP and its gene (GC) which has three common allelic variants-GC*1F, GC*1S and GC*2 [8, 9, 47]-playing a significant role in the pathogenesis of lung diseases [39]. These polymorphisms can alter VDBP structure, circulating levels, and ligand-binding affinity, thereby modulating the transport and bioavailability of vitamin D metabolites [9]. However, the precise mechanisms remain controversial [48]. Upon integrating the results of 30 articles, we found that the GC*1F allele may increase the risk of COPD in East Asian populations, while the GC*2 allele appears to confer a protective effect against COPD, particularly in non-East Asian populations. Conversely, GC*2 may increase the incidence of asthma(*P* ≤ 0.001). This variation suggests that genetic background, as well as potential environmental modifiers, may influence the impact of VDBP polymorphisms on disease risks.

The relationship between rs7041 single nucleotide polymorphisms and the risk of COPD has been extensively studied [8, 49]. Previous research has demonstrated significant associations between the VDBP gene rs7041 polymorphism, the risk of COPD and changes in circulating 25-hydroxyvitamin-D concentrations [7]. However, our meta-analysis found no association between the rs7041 and rs4588 single nucletide polymorphisms and COPD risk. Similarly, the meta-analysis conducted by Yang et al. found no association between rs7041 and COPD risk [7]. Therefore, further investigation is still needed on the association between rs4588 and COPD. In the present study, the GC*1F allele was identified as a risk factor for COPD, consistent with previous research results [14, 49, 50]. Specifically, among East Asian populations, individuals carrying the GC*1F/1F genotype exhibit a higher risk of COPD, while this association is not significant in non-East Asians. This may reflect underlying differences in population genetic structure, environmental risk factors, smoking behavior, and methodological characteristics among studies.

The biological differences among the major VDBP isoforms may partially explain the observed disease associations. Previous studies have suggested that GC*1F, GC*1S, and GC*2 differ in circulating VDBP concentrations, affinity for vitamin D metabolites, and downstream immunological functions [10]. The GC*2 variant is less able to activate macrophages, for the absence of a glycosylated residue at position 420 [12, 28, 47], so it plays a protective role in COPD. However, GC*1F retains the glycosylation site at position 420, which may influence macrophage activation and inflammatory responses [14]. In addition, circulating VDBP may enhance the neutrophil chemotactic activity of complement derived C5a, which is associated with inflammation [14].These mechanisms may provide a plausible explanation for the increased COPD susceptibility associated with GC*1F.

Conversely, the GC*2 allele may act as a risk factor for asthma [34, 37]. This finding is consistent with some previous studies which reported an increased susceptibility to asthma among GC*2 allele [37, 51]. However, unlike our results, one study found no significant association between GC*2 and asthma risk [49]. A possible reason for the increased asthma risk is that GC*2 may impair vitamin D-mediated immune tolerance, antimicrobial defense, and regulation of Th_2_ inflammation, thereby increasing susceptibility to asthma. The different inflammation pathways may explain the opposite role of GC*2 in COPD and asthma. This discrepancy highlights the need for further research to elucidate the relationship between asthma and VDBP polymorphisms [52].

Additionally, the G allele of rs7041 has been associated with an increased susceptibility to COVID-19 [53], potentially due to its role in reducing the affinity of VDBP for vitamin D, which is consistent with the findings of Ozturk et al. [42]. This diminished binding affinity may compromise immunoregulatory and tissue-protective functions, thereby heighten the risk of severe illness during cytokine storms [40–42]. In tuberculosis studies, there are conflicting conclusions regarding the association between rs7041/rs4588 polymorphisms and disease risk. Some research suggests that the rs4588 may confer a protective effect under specific dominant models [44], while the rs7041 AA + AC genotype may increase susceptibility to the disease [54]. But Talat et al. [55] reported that vitamin D deficiency was associated with a fivefold increased risk of progression to active tuberculosis [56]. Owing to the limited number of eligible studies, a formal meta-analysis could not be conducted; however, descriptive synthesis suggested consistent associations between COVID-19 or tuberculosis and VDBP. Therefore, further large-scale, well-designed studies are warranted to clarify these associations.

Several limitations of this study should be taken into account when interpreting these findings. Firstly, the number of studies included on COVID-19 and tuberculosis was comparatively small, which may affect the reliability and generalizability of the results. Secondly, substantial heterogeneity was observed in several analyses. This may reflect the differences in study design, genotyping methods, control selection, and population characteristics. Moreover, the broad classification into East Asian and non-East Asian populations may have obscured population-specific associations among genetically and environmentally diverse groups. Thirdly, due to the lack of sufficient clinical data, we were unable to conduct stratified analyses for confounding factors such as age, gender, smoking exposure, vitamin D status, or other environmental risk factors were performed, which may have missed important influencing factors. Fourthly, publication bias could not be formally assessed in analyses involving fewer than 10 studies and therefore cannot be excluded. Fifthly, differences in recruitment strategies, diagnostic criteria, and genotyping technologies between older and more recent studies may have introduced ascertainment bias. Sixthly, although five major databases were systematically searched and reference lists were manually screened, restricting the search to English- and Chinese-language publications may have introduced language bias. Finally, multiple comparisons across genetic models, diseases and ethnic subgroups increased the possibility of chance-positive findings.

In summary, no association was found between rs7041/rs4588 and COPD risk. However, the GC*1F allele may be associated with susceptibility to COPD in East Asian populations. The GC*2 allele may associate with reduced risk for COPD, with this protective effect being more significant in non-East Asian populations, while increase the risk of asthma. GC*1S may have a protective tendency to asthma. Thus, future studies should focus on whether GC genotypes can predict responses to vitamin D supplementation, anti-inflammatory therapy, or treatments targeting VDBP-related pathways. Evidence for COVID-19 and tuberculosis remains insufficient for firm conclusions. Given the limited sample sizes and heterogeneity in outcome definitions across included studies, our findings should be considered preliminary and await validation in prospective, adequately powered investigations that employ uniform diagnostic criteria.

This study was funded by the Natural Science Foundation of Shandong Province (General Program ZR2021MH359 and Youth Program ZR2022QH371). But there isn’t any competing interests.

## Supplementary Information

Supplementary Material 1.

Supplementary Material 2.

## Data Availability

All data supporting the findings of this study are available within the paper and its Supplementary Information. Microsatellite primer sequences are provided in Supplementary Table 2, along with original reference describing the microsatellites used in this study.

## Abbreviations

COPD: Chronic obstructive pulmonary disease
COVID-19: Corona Virus Disease 2019
GWAS: Genome-wide association studies
VDBP: Vitamin D binding protein
SNP: Single nucleotide polymorphism
CNKI: Chinese National Knowledge Infrastructure
FEV_1_: Forced expiratory volume in the first second
FEV_1_%: Percentage predicted values for FEV_1_
FEV_1_/FVC: Forced expiratory volume in one second/ forced vital capacity
NOS: Newcastle Ottawa Scale
OR: Odds ratio
95%CI: Confidence interval
M-H method: Mantel–Haenszel method
