# Supplementary material for: Vitamin D binding protein gene polymorphisms and the risk of respiratory diseases: a meta-analysis and systematic review
Source: BMC Med Genomics. 2026 Aug 13;19:175. doi: 10.1186/s12920-026-02443-1 (PMC13757158; doi:10.1186/s12920-026-02443-1)
Supplement: Supplementary file 1 [file 12920_2026_2443_MOESM1_ESM.docx]

**Supplementary file**

**Vitamin D binding protein gene polymorphisms and the risk of respiratory disease: a meta-analysis and systemetic review**

Jing Zhang^1†^，Hongwen Li^1†^, Xiangyu Chi^1^, Guoying Zhang^2#^, Hongjuan Jiang^1^*

Figure S1:Conflation of SNP-level and isoform-level analyses

Table 4: Detailed search strategy

| Detailed Search Strategy | | | | | | |
| --- | --- | --- | --- | --- | --- | --- |
| **Pubmed** |  |  |  |  |  |  |
| ("vitamin D binding protein"[Title/Abstract] |  |  |  |  |  |  |
| OR "GC globulin"[Title/Abstract] |  |  |  |  |  |  |
| OR VDBP[Title/Abstract]) |  |  |  |  |  |  |
|  |  |  |  |  |  |  |
| AND |  |  |  |  |  |  |
|  |  |  |  |  |  |  |
| ("respiratory disease"[Title/Abstract] |  |  |  |  |  |  |
| OR COPD[Title/Abstract] |  |  |  |  |  |  |
| OR "chronic obstructive pulmonary disease"[Title/Abstract] |  |  |  |  |  |  |
| OR asthma[Title/Abstract] |  |  |  |  |  |  |
| OR tuberculosis[Title/Abstract] |  |  |  |  |  |  |
| OR COVID-19[Title/Abstract]) |  |  |  |  |  |  |
|  |  |  |  |  |  |  |
| **CNKI** |  |  |  |  |  |  |
| "vitamin D binding protein" |  |  |  |  |  |  |
| "GC globulin" |  |  |  |  |  |  |
| OR "VDBP") |  |  |  |  |  |  |
|  |  |  |  |  |  |  |
| AND |  |  |  |  |  |  |
|  |  |  |  |  |  |  |
| ("chronic obstructive pulmonary disease" |  |  |  |  |  |  |
| OR COPD |  |  |  |  |  |  |
| OR asthma |  |  |  |  |  |  |
| OR COVID-19 |  |  |  |  |  |  |
| OR tuberculosis) |  |  |  |  |  |  |
|  |  |  |  |  |  |  |
| **Wan Fang** |  |  |  |  |  |  |
| ("vitamin D binding protein" |  |  |  |  |  |  |
| OR "GC globulin" |  |  |  |  |  |  |
| OR "VDBP") |  |  |  |  |  |  |
|  |  |  |  |  |  |  |
| AND |  |  |  |  |  |  |
|  |  |  |  |  |  |  |
| ("chronic obstructive pulmonary disease " |  |  |  |  |  |  |
| OR COPD |  |  |  |  |  |  |
| OR asthma |  |  |  |  |  |  |
| OR COVID-19 |  |  |  |  |  |  |
| OR tuberculosis) |  |  |  |  |  |  |
|  |  |  |  |  |  |  |
| **Web of Science** |  |  |  |  |  |  |
| TS= |  |  |  |  |  |  |
| "vitamin D binding protein" |  |  |  |  |  |  |
| "GC globulin" |  |  |  |  |  |  |
| VDBP |  |  |  |  |  |  |
| "group-specific component" |  |  |  |  |  |  |
|  |  |  |  |  |  |  |
| AND |  |  |  |  |  |  |
|  |  |  |  |  |  |  |
| "respiratory disease" |  |  |  |  |  |  |
| OR "respiratory diseases" |  |  |  |  |  |  |
| OR COPD |  |  |  |  |  |  |
| OR "chronic obstructive pulmonary disease" |  |  |  |  |  |  |
| OR asthma |  |  |  |  |  |  |
| OR tuberculosis |  |  |  |  |  |  |
| OR TB |  |  |  |  |  |  |
| OR COVID-19 |  |  |  |  |  |  |
| OR "Coronavirus Disease 2019" |  |  |  |  |  |  |
|  |  |  |  |  |  |  |
| **Embase** |  |  |  |  |  |  |
|  |  |  |  |  |  |  |
| 'vitamin d binding protein'/exp |  |  |  |  |  |  |
| OR 'vitamin d binding protein':ti,ab |  |  |  |  |  |  |
| OR 'gc globulin':ti,ab |  |  |  |  |  |  |
| OR vdbp:ti,ab |  |  |  |  |  |  |
| OR 'group specific component':ti,ab |  |  |  |  |  |  |
|  |  |  |  |  |  |  |
| AND |  |  |  |  |  |  |
|  |  |  |  |  |  |  |
| 'respiratory tract disease'/exp |  |  |  |  |  |  |
| OR 'chronic obstructive lung disease'/exp |  |  |  |  |  |  |
| OR 'asthma'/exp |  |  |  |  |  |  |
| OR 'tuberculosis'/exp |  |  |  |  |  |  |
| OR 'covid-19'/exp |  |  |  |  |  |  |
| OR 'respiratory disease':ti,ab |  |  |  |  |  |  |
| OR copd:ti,ab |  |  |  |  |  |  |
| OR 'chronic obstructive pulmonary disease':ti,ab |  |  |  |  |  |  |
| OR asthma:ti,ab |  |  |  |  |  |  |
| OR tuberculosis:ti,ab |  |  |  |  |  |  |
| OR tb:ti,ab |  |  |  |  |  |  |
| OR covid-19:ti,ab |  |  |  |  |  |  |
| OR 'coronavirus disease 2019':ti,ab |  |  |  |  |  |  |
|  |  |  |  |  |  |  |
| AND |  |  |  |  |  |  |
|  |  |  |  |  |  |  |
| [humans]/lim |  |  |  |  |  |  |
|  |  |  |  |  |  |  |
| AND |  |  |  |  |  |  |
|  |  |  |  |  |  |  |
| (english OR chinese) |  |  |  |  |  |  |
|  |  |  |  |  |  |  |
| Date |  |  |  |  |  |  |
| from January 1990 to March 2026. |  |  |  |  |  |  |
