# Supplementary material for: Vitamin D binding protein gene polymorphisms and the risk of respiratory diseases: a meta-analysis and systematic review
Source: BMC Med Genomics. 2026 Aug 13;19:175. doi: 10.1186/s12920-026-02443-1 (PMC13757158; doi:10.1186/s12920-026-02443-1)
Supplement: Supplementary file 2 [file 12920_2026_2443_MOESM2_ESM.docx]

PRISMA 2020 Checklist

Manuscript title: Vitamin D binding protein gene polymorphisms and the risk of respiratory disease: a meta-analysis

| Section and Topic | Item # | Checklist Item | Location (page) |
| --- | --- | --- | --- |
| ****TITLE**** | 1 | Vitamin D binding protein gene polymorphisms and the risk of respiratory disease: a meta-analysis and systematic review | Title page |
| ****ABSTRACT**** | 2 | See the PRISMA 2020 for Abstracts checklist. | PRISMA 2020 for Abstracts checklist(Appendix 2) |
| ****INTRODUCTION**** |  |  |  |
| Rationale | 3 | Describe the rationale for the review in the context of existing knowledge. | Introduction, paragraphs 1–2 |
| Objectives | 4 | Provide an explicit statement of the objective(s) or question(s) the review addresses (e.g., PICO). | Last paragraph of Introduction |
| ****METHODS**** |  |  |  |
| Eligibility criteria | 5 | Specify the inclusion and exclusion criteria for the review (e.g., PICO, study design). | Methods: Inclusion and exclusion criteria(page 8) |
| Information sources | 6 | Specify all databases, registers, websites, organisations, and other sources searched. Give the date when each source was last searched. | Methods: Search strategy (searched up to March 2026)(page 7) |
| Search strategy | 7 | Present the full search strategies for all databases, registers and websites, including any filters and limits used. | Methods: Search strategy(page 7) and the supplementary table 4 |
| Selection process | 8 | Specify the methods used to decide whether a study met the inclusion criteria of the review. | Methods: Data extraction(page 8) |
| Data collection process | 9 | Specify the methods used to collect data from reports, including how many reviewers did this. | Two investigators independently extracted data. |
| Data items | 10 | List and define all outcomes for which data were sought. Specify how missing data were handled. | Methods: Data extraction + Table 1(page 8 and table 1) |
| Study risk of bias assessment | 11 | Specify the methods used to assess risk of bias in the included studies. | Methods: Quality score assessment (NOS scale)(page 9 and table 2) |
| Effect measures | 12 | Specify for each outcome the effect measure(s) (e.g., OR, RR, MD). | Methods: Statistical analysis (OR with 95%CI) |
| Synthesis methods | 13 | Describe the methods used to synthesise results (e.g., fixed/random effects model, heterogeneity handling). | Methods: Statistical analysis (Heterogeneity assessment, subgroup analyses, sensitivity analyses, and genetic-model selection) |
| Reporting bias assessment | 14 | Describe the methods used to assess the possibility of reporting bias (e.g., Egger’s test, funnel plots). | Methods: Statistical analysis (Egger's test when applicable)(page 8-9) |
| Certainty assessment | 15 | Describe the methods used to assess certainty (or confidence) in the body of evidence (e.g., GRADE). | Not explicitly described (optional) |
| ****RESULTS**** |  |  |  |
| Study selection | 16 | Describe the results of the search and selection process, ideally with a flow diagram. | Results: Figure 1 |
| Study characteristics | 17 | Cite each included study and present its characteristics (e.g., sample size, population, genotypes). | Results: Table 1 |
| Risk of bias in studies | 18 | Present assessments of risk of bias for each included study. | Quality assessment using the Newcastle–Ottawa Scale (Table 2) |
| Results of individual studies | 19 | For all outcomes, present for each study: summary statistic (e.g., OR) and confidence interval. | Results text + forest plots (Figures 2–8)(page 12-18) |
| Results of syntheses | 20 | Present the results of each synthesis, including confidence intervals and measures of heterogeneity. | Results subsections + forest plots(page 12-18 and figure 1-8) |
| Reporting biases | 21 | Present assessments of reporting bias (e.g., Egger’s test P values). | Results: Publication bias analyses(Egger's test performed where sufficient studies were available)(page 23，line 467-470) |
| Certainty of evidence | 22 | Present assessments of certainty (or confidence) in the body of evidence (e.g., GRADE). | Not reported (optional) |
| ****DISCUSSION**** |  |  |  |
| Discussion | 23 | Provide a general interpretation of the results in the context of other evidence, and discuss limitations. | Discussion, paragraphs 1–5(page 19-22) |
| Limitations | 24 | Discuss limitations of the evidence and of the review process. | Discussion, paragraph 6(page 22-23) |
| Conclusions | 25 | Provide a general interpretation of the results and implications for future research. | Last paragraph of Discussion(page 23) and the abstract |
| ****OTHER INFORMATION**** |  |  |  |
| Registration and protocol | 26 | Provide registration information (e.g., PROSPERO) or state that the review was not registered. | Not registered |
| Support | 27 | Describe sources of financial or non-financial support for the review. | Funding statement + last paragraph of Discussion(line 490-492) |
| Competing interests | 28 | Declare any competing interests of review authors. | Declarations: Competing interests(page 26) |
| Availability of data, code, etc. | 29 | Report whether and how the data, analytic methods, etc. can be accessed. | Declarations: Availability of data and materials(page 26) |
